# A Review of Resveratrol as a Potent Chemoprotective and Synergistic Agent in Cancer Chemotherapy

**DOI:** 10.3389/fphar.2018.01534

**Published:** 2019-01-09

**Authors:** Qicai Xiao, Wangshu Zhu, Wei Feng, Su Seong Lee, Albert Wingnang Leung, Jun Shen, Liqian Gao, Chuanshan Xu

**Affiliations:** ^1^School of Pharmaceutical Sciences (Shenzhen), Sun Yat-sen University, Guangzhou, China; ^2^School of Chinese Medicine, Faculty of Medicine, The Chinese University of Hong Kong, Shatin, Hong Kong; ^3^Department of Radiology, Sun Yat-sen Memorial Hospital, Sun Yat-sen University, Guangzhou, China; ^4^Institute of Bioengineering and Nanotechnology, Singapore, Singapore; ^5^Division of Chinese Medicine, School of Professional and Continuing Education, The University of Hong Kong, Pokfulam, Hong Kong; ^6^Key Laboratory of Molecular Target and Clinical Pharmacology, State Key Laboratory of Respiratory Disease, School of Pharmaceutical Sciences and Fifth Affiliated Hospital, Guangzhou Medical University, Guangzhou, China; ^7^Shenzhen Research Institute, The Chinese University of Hong Kong, Shenzhen, China

**Keywords:** natural products, resveratrol, cancer, chemotherapy, side effects

## Abstract

**Background:** Cancer has become a major disease endangering human health around the world. Conventional chemotherapy suffers from many side effects including pain, cardiotoxicity, hepatotoxicity, and renal toxicity. This review aims to describe a natural product of resveratrol as a chemoprotective and synergistic agent in the modulation of cancer chemotherapy.

**Methods:** The publications were identified by comprehensive searching of SciFinder, PubMed, Web of Science, and our own reference library. Search terms included combinations of “resveratrol,” “cancer,” “natural products,” “chemotherapy,” and “side effects.” Selection of material focused on resveratrol reducing the side effects on cancer chemotherapy.

**Results:** Thirty one references were referred in this review to outline resveratrol as a potent chemoprotective and synergistic agent in cancer chemotherapy, including 22 papers for describing the chemoprotective effects, and 9 papers for illustrating the synergistic effects.

**Conclusion:** This study provides a systematic summary of resveratrol serving as a potent chemoprotective and synergistic agent to reduce the associated-side effects and enhance the therapeutic outcomes in cancer chemotherapy. Further studies in terms of resveratrol on a large amount of preclinical tests and clinical trials are highly demanded.

## Introduction

Cancer, also known as malignant tumor, is a major public health problem around the world, not only brings unutterable pains to patients, but also leads to heavy economic burden to the families and society (Chen et al., 2016; Siegel et al., 2016, 2017). The global incidence of cancer is increasing yearly, and the total mortality has exceeded many other diseases, making it become a great killer to human health. It is reported that there was about 90.5 million cancer patients in 2015, and almost 14.1 million new cases occur each year, excluding skin cancer but melanoma (Bernard and Christopher, 2014; Vos et al., 2016). The global cancer death was around 8.8 million in 2015, which is almost 1 in 6 of all global deaths (Vos et al., 2016). In spite of tremendous efforts having been paid to improve this situation and find novel treatments, such as, radiotherapy, surgery, and immunotherapy, chemotherapy is up to now still the major approach used in clinic. However, chemotherapy often suffers from many side effects, such as cardiotoxicity, hepatotoxicity, renal toxicity and, easy recurrence and metastasis (Monsuez et al., 2010; Shukla et al., 2011).

Natural products have been one of the most important and essential sources in drug discovery and development (Shen, 2015; Wang et al., 2016; Xiao et al., 2018). For years, humans have relied on natural products and/or natural herbal formulations for the maintenance of health, prevention of diseases, and improvement of mental and physical health. Particularly, many studies have demonstrated that some natural products in combination with chemotherapeutic agents can play chemoprotective and/or synergistic effects in terms of reducing cancer chemotherapy-associated side effects and enhancing the therapeutic efficacy (Cragg and Pezzuto, 2016).

**Figure 1:**
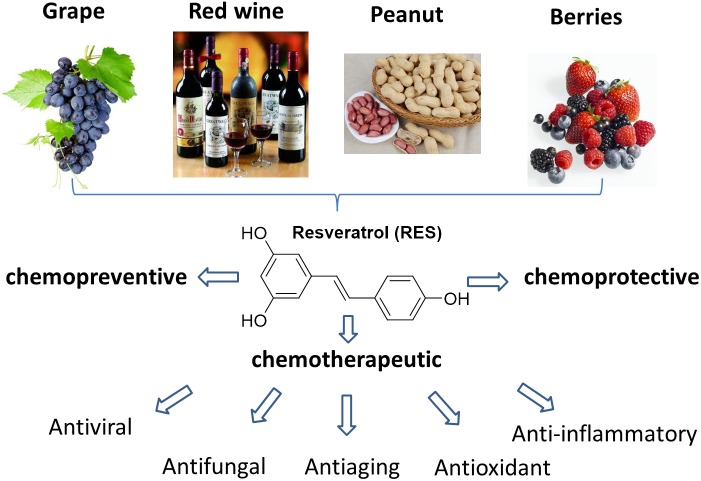
Resources of RES and its potent biological activities.

Resveratrol (RES, 3,5,4′-trihydroxy-*trans*-stilbene) is a naturally occurring polyphenol presented in lots of dietary substances, such as grapes, wine, nuts, berries and many other human foods (Figure 1) (Berman et al., 2017). It often occurs as a white powder with moderate water solubility (0.03 mg/mL). The molecular skeleton of RES is composed by two phenolic rings, one with a *para* hydroxyl group, and the other with an *ortho* double hydroxyl groups. The two benzene rings are connected through a double bond that affords isomers with *cis* and *trans* configuration. Usually, the most referred RES is the *trans* isomer, which is the most abundant and biologically active compound. It is reported that the total content of RES is around 50–100 μg/g in fresh grape skin, 5.1 μg/g in boiled peanuts, 0.31 μg/g in peanut buffer and 0.98–1.80 mg/L in red wine (Burns et al., 2002; Cal et al., 2003). Besides, a large amount of RES is also found in Itadori plants and tea, and the commercial grape juice contains about 4 mg/L of RES (Table 1) (Burns et al., 2002).

**Table 1:** Contents of RES in different substances.

Sample	Approximate amount of RES
Grape skin (fresh)	50–100 μg/g
Peanuts (raw)	0.07–1.8 μg/g
Peanuts (boiled)	1.8–7.1 μg/g
Peanut buffer	0.16–0.5 μg/g
Red wine	0.98–1.80 mg/L
Itadori leaf (young)	867 ± 17 μg/g
Itadori leaf (old)	370 ± 9 μg/g
Itadori stem (young)	497 ± 4 μg/g
Itadori stem (old)	83 ± 3 μg/g
Itadori root (commercial)	2170 ± 9 μg/g
Itadori tea	974 ± 2 μg/g

Resveratrol as a natural polyphenol, it has been confirmed to have a broad range of biological activities, including anti-inflammatory, anti-oxidant, anti-viral, anti-fungal and anti-aging effects (Baur et al., 2006; Carter et al., 2014; Kotecha et al., 2016; Chedea et al., 2017; Nawaz et al., 2017). The broad biological activities of RES is primarily ascribed to its unique structure character with multiple phenolic hydroxyl groups, as polyphenol compounds are capable of scavenging free radicals to form more stable molecules with less toxicity than the original radicals (Frémont, 2000). In 1997, a pioneering work initiated by Jiang et al. revealed that RES could inhibit the cellular effects involving the three stages of carcinogenesis, including tumor initiation, promotion and progression, arousing the impetus and interest in the investigation of RES (Jang et al., 1997).

Numerous studies have shown that RES possessed chemoprotective effects, such as cardioprotective activity and neuroprotective activity (Cho et al., 2017; Riba et al., 2017; Sarubbo et al., 2017; Cai et al., 2018). Upon co-administration with chemotherapeutic agents, RES could decrease the associated-side effects while enhance the therapeutic efficacy concomitant with cancer chemotherapy. Currently, a few papers summarized the therapeutic benefits and chemopreventive effects of RES, ranging from autoimmune diseases to neoplastic diseases (Aziz et al., 2003; Kotecha et al., 2016; Berman et al., 2017; de Brito Monteiro et al., 2017; Ko et al., 2017). To the best of our knowledge, there is up to now no review article focusing on RES as a combinational agent to reduce the associated-side effects and enhance the therapeutic efficacy in cancer chemotherapy. Therefore, this work represents the first survey and summary of RES as a chemoprotective and synergistic agent in cancer chemotherapy. In addition, the mechanisms of RES-medicated cancer therapy, potential limitations and future perspectives of RES in clinical applications are also presented and discussed.

## Materials and Methods

### Search Strategy and Information Sources

This review was in line with recommendations from the Preferred Reporting Items for Systematic Reviews and Meta-Analyses (PRISMA) statement. The publications were identified by comprehensive searching of SciFinder, PubMed, Web of Science, and our own reference library. Search terms included combinations of “resveratrol,” “cancer,” “natural products,” “chemotherapy,” and “side effects.”

### Study Selection, Data Collection, and Exclusion/Inclusion Criteria

Selection of material was limited to papers published in English language. All of the publications were at least checked by two investigators. Patents, books, dissertations, and abstracts of conferences were excluded. Also the studies with regard only to the therapeutic effects of RES such as anti-aging, anti-fungal, anti-inflammatory, and anti-viral activities were excluded. Studies related to RES on reducing chemotherapy-induced side effects, the chemoprotective effects of RES against external factors such as carcinogens-induced damages, and the synergistic effects of RES to enhance the therapeutic efficacy are the inclusion criteria.

## Results

**Figure 2:**
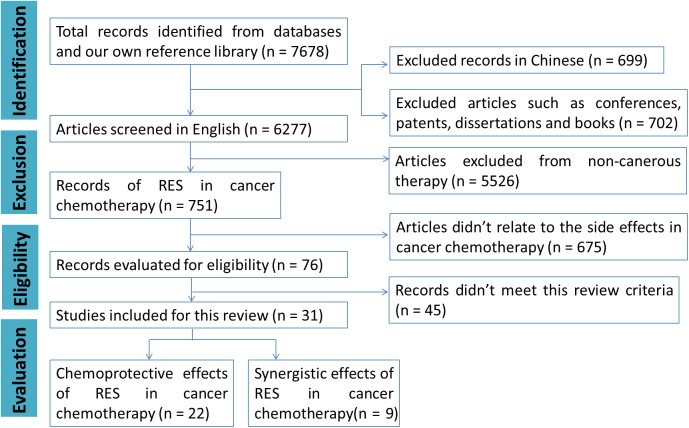
The flow chart of publications screening process.

Using the search strategies mentioned above afforded 7,678 records, and among which 699 records were in Chinese. After applying the exclusion criteria, 7,647 publications were removed, which includes; (a) 702 records, such as conferences, patents, dissertations and books, (b) 5,526 records were not correlated to cancer chemotherapy, (c) 675 records did not refer to the side effects in cancer chemotherapy, and (d) 45 records did not meet the inclusion criteria. A flow chart (Figure 2) illustrates the screening process and study selection.

### RES as a Chemoprotective Agent in Cancer Chemotherapy

The adverse effects induced by chemotherapeutic agents are always the obstacles for their broad application in clinic. As a naturally occurring multifunctional molecule, RES has been reported to be capable of performing protective effects to reduce the associated-side effects induced by chemotherapeutic drugs.

#### Cardio-Protective Effects in Cancer Chemotherapy

Cardiotoxicity is a common side effect induced by chemotherapeutic agents in cancer chemotherapy. The generally used ANT, such as Dox or daunorubicin, usually brings severely cardiotoxic effects including myocarditis, arrhythmias, dilated cardiomyopathy, and congestive heart failure (Chatterjee et al., 2010). It is accepted that the production of free radicals and oxidative stress are involved in cardiotoxicity, and Dox was found to enhance the formation of ROS in heart tissues (Šimůnek et al., 2009; Deavall et al., 2012). Sheu et al. (2015) investigated the anti-oxidizing effects of 4 different kinds of antioxidants including RES. It was found that the intracellular ROS levels were significantly decreased after being co-treated with the antioxidants and Dox. In addition, the SOD levels that involved in eliminating ROS were increased. Among these tested antioxidants, RES was found to be the best one to neutralize ROS. Therefore, RES can be used as an efficient supplement to reduce the cardiotoxicity in Dox-mediated cancer chemotherapy. Besides, some other studies showed that the adverse effects of ANT might be ascribed to the metabolites of ANT that is generated by a two electron reduction, such as DOXol, a reduced metabolite from Dox, which is more cardiotoxic than Dox (Hanna et al., 2014; Piska et al., 2017). The reason for the high cardiotoxicity of DOXol is that it acts as a potent inhibitor of Ca^2+^, Mg^2+^, and Na^+^/K^+^-ATPases, and Ca^2+^ is the link between electric stimulation and cell contraction. Disruption of the calcium regulation system is considered to be responsible for DOXol cardiotoxicity (Piska et al., 2017). In general, the generation of ANT metabolites is catalyzed by cytosolic enzymes of CBR and AKR. Therefore, the inhibitors for CBR and AKR are believed to have cardioprotective effects (Huang et al., 2010; Zhou et al., 2015). Ito et al. (2013) reported that RES and its analogs (4′-methoxy, 5-methoxy and 4′-amino) could bind CBR1 to inhibit its activity, thus reducing the cardiotoxicity of Dox.

In addition, another kind of traditional anticancer drug named As_2_O_3_ is mainly used for the treatment of acute APL, hematologic malignancies and some solid tumors (Waxman and Anderson, 2001; Emadi and Gore, 2010). The adverse events of As_2_O_3_ include cardiotoxicity, electrocardiogram abnormalities, nephrotoxicity, hepatotoxicity, APL differentiation syndrome, and sudden death (Westervelt et al., 2001; Vuky et al., 2002; Emadi and Gore, 2010). Just as ANT-induced side effects, the primary pathogenic mechanism of As_2_O_3_ is correlated with the oxidative stress, which engenders the dysfunction of antioxidant defense system, thus leading to oxidative damage to cellular macromolecules due to the generation of free radicals (Emadi and Gore, 2010; Wang et al., 2012). Zhao et al. (2008) reported that prior treatment with RES (3 mg/kg) 1 h before As_2_O_3_ administration could protect against As_2_O_3_-induced cardiotoxicity, and attenuate myocardial injury, DNA fragmentation and oxidative damage.

#### Nephroprotective Effects in Cancer Chemotherapy

It is believed that ingestion of arsenic, both from water supplies and foods could cause renal damage, and chronic arsenic exposure could cause degenerative changes in kidneys (Liu et al., 2000). Moreover, As_2_O_3_ treatment for relapsed or refractory APL and MM were also reported to cause renal injury, such as elevated serum creatinine, blood urea nitrogen and protein urea concentration levels in clinical studies (Zhang et al., 2000). Yu et al. (2013) found that RES could facilitate the methylation and excretion of ascetic, thus significantly attenuated As_2_O_3_-induced arsenic accumulation, structural abnormalities and arsenic-related toxicity in kidney.

#### Hepaticprotective Effects in Cancer Chemotherapy

Liver plays a significant role in clearing and transforming chemicals, which is susceptible to the toxicity from these toxic agents. Besides cardiotoxicity and nephrotoxicity, it was also reported that As_2_O_3_ could cause hepatotoxicity which is associated with the generation of ROS. Zhang et al. (2013) reported that RES could attenuate As_2_O_3_-induced oxidative stress, decrease the arsenic accumulation in liver, and increase the activities of antioxidant enzymes, thus preventing As_2_O_3_-induced hepatotoxicity. In addition, several other studies reported that RES could protect acetaminophen-induced and pyrogallol-induced hepatotoxicity through its potent antioxidant activities (Şener et al., 2006; Upadhyay et al., 2008).

#### Gastrointestinal Protective Effects in Cancer Chemotherapy

The adverse effects in gastrointestinal tract are also present in chemotherapeutic drug mediated therapy, including anorexia, nausea, vomiting, constipation and diarrhea. Aspirin is a typical NASID widely used for the treatment of inflammation, fever and pain for more than one century. It functions through inhibiting the COX - an intracellular enzyme that catalyzes the rate-limiting step in the metabolic conversion of arachidonic acid to PGs and related eicosanoids (Patrono et al., 2005). Recent studies indicated that aspirin can be used in cancer prevention, particularly for reducing the risk of colorectal neoplasms (Chan et al., 2007; Thun et al., 2012; Thorat and Cuzick, 2013). However, the primary side effects in aspirin-mediated therapy are the gastric ulceration, which is due to the inhibition of COX-1. Dey et al. (2009) reported that a lower dose of RES (2 mg/kg) has the capability to protect gastric mucosa from NSAID-induced side effects, while a higher dose of RES (10 mg/kg) demonstrated a contraindicative effect, which might be resulted from the balance level between COX-1 and eNOS. To further ameliorate the side effects caused by aspirin, Zhu et al. (2015) constructed the RAH, which acted as a prodrug with the capability of releasing aspirin and RES simultaneously. It was found that most of RAH was transited out from stomach, while parts of the intact RAH were detected in the mouse colon tissue collected at 1 and 2 h after administration. The stability tests of RAH in cancer cells revealed that RAH was hydrolyzed into deacetyl-RAH and subsequently decomposed to release RES and aspirin over 24 h of incubation, suggesting that RAH is able to bear the acidic environment in stomach, avoid enzymatic hydrolysis in small intestine, and incorporate into the colon tissue to perform its antitumor activity. Another study initiated by Ko et al. (2016) showed that pretreatment wistar rats with whole grape juice that contains large amounts of RES could induce gastric emptying, improve the tubular dilatation and tubular cell vacuolization in renal tubules, thus bringing beneficial effects in preventing cisplatin-induced gastrointestinal tract disorders.

#### Protective Effects in UVR-Induced Skin Cancer

Skin serves lots of essential functions including protection from environmental stressors and infections. Furthermore, it also provides immunological surveillance against germs, allergens, and irritants attempting to enter the body (Di Meglio et al., 2011; Draelos, 2012). UVR can induce cell death through an apoptotic pathway, demonstrating certain therapeutic potential (Ji et al., 2015). However, UVR exposure is also regarded as one of the dominant causes for skin cancer (65–90%), in addition to the genetic and environmental factors (Bowden, 2004; Lucas et al., 2008). A large number of studies indicated that RES is able to protect against UVR-induced skin cancer via modulating the proteins that are involved in apoptosis (Reagan-Shaw et al., 2004; Aziz et al., 2005a; Ndiaye et al., 2011). Jang et al. (1997) revealed that topical application of RES could afford substantially protective effects against chemically induced skin carcinogenesis in CD-1 mice. Afaq et al. (2003) reported that topical application of RES could inhibit UV-B-induced skin edema and cause a significant decrease in UV-B-mediated hydrogen peroxide production and the infiltration of leukocytes in SKH-1 hairless mice. It is reported that long term topical application of RES, both pre-treatment and post-treatment, could inhibit the tumor incidence and delay in the onset of tumorigenesis, (Aziz et al., 2005b) while the short term topical application of RES could lead to a marked inhibition of cellular proliferation and phosphorylation of surviving (Aziz et al., 2005a). Additionally, a few studies showed that some cell cycle regulatory molecules, inhibiting factors of apoptotic proteins, and cell signaling pathways under RES-mediated chemoprotective effects are also involved against the adverse effects of UVR-induced photocarcinogenesis (Adhami et al., 2003; Reagan-Shaw et al., 2004).

#### Protective Effects Against Carcinogenic Substances-Induced Damage

In addition to the chemoprotective effects during cancer chemotherapy, RES can also exert its protective effects against the toxicity induced by carcinogenic substances. For instance, Revel et al. (2003) found that RES (50 mg/kg/week) could protect the lung from DNA damage and apoptosis that is resulted from the carcinogen of benzo[a]pyrene. Tessitore et al. (2000) found that upon administration of RES (200 mg/kg/day), the number of aberrant crypt foci in azoxymethane-induced colon carcinogenesis in rats was reduced. Tang et al. (2009) found that RES treatment could partially prevent carbon tetrachloride-induced acute liver damage. Additionally, in a DMBA-induced mammary carcinogenesis model in rats, dietary administration of RES had indeed no effects on tumor volume but produced striking reductions in the incidence and multiplicity, and thus extended the latency period in tumor development (Banerjee et al., 2002).

### RES as a Synergistic Agent in Cancer Chemotherapy

RES modulates multiple pathways involved in cell cycle, apoptosis and inflammation. In addition to the chemopreventive and chemoprotective effects, RES also demonstrates potent anticancer activity (Sarkar et al., 2009). It is known that a single treatment often demonstrates weak activity, incomplete efficiency, and drug-resistance. The combination therapy recently developed through simultaneously combining more than two drugs, often brings better therapeutic outcomes.

Estrogens as natural hormones, are mediated by ERs. Exposure to estrogens has been recognized as a major risk for a variety of cancers, especially for breast cancer and other estrogen-mediated cancers (Henderson and Feigelson, 2000; Cavalieri and Rogan, 2014). The major cause for this observation is due to the formation of estrogen-DNA adducts that initiate tumorigenesis (Pruthi et al., 2012). It was found by Zahid et al. (2007) that NAcCys and RES are efficient inhibitors for the formation of estrogen-DNA adducts. The further study showed that upon treatment of MCF-10F cells with RES and NAcCys simultaneously, the formed estrogen-DNA adducts decreased more than those treated by the individual compound alone, and in the meantime the tumor initiation process is more efficiently disrupted (Zahid et al., 2011). Wu et al. (2004) found that RES can enhance the anti-tumor effect of 5-FU on murine hepatoma model *in vitro* through inducing the S phase arrest of H22 cells. A similar study initiated by Dun et al. (2015) also showed that RES could enhance the anti-tumor efficacy of 5-FU by inducing S-phase arrest both *in vitro* and *in vivo*.

Action of RES alone or in combination with ROSC-a CDK inhibitor, on cell cycle progression in human HL-60 leukemia cells was studied by Komina and Wȩsierska-Gądek (2008). A striking synergistic effect was observed after a combined treatment for 24 h and followed by sequential post-incubation for 48 h in the presence of RES. The G(1) cell population was increased up to 80% at a fourfold lower dose of ROSC with the addition of RES (Komina and Wȩsierska-Gądek, 2008).

In another study, Casanova and his co-workers investigated the combined action of RES with MEL, a well-known alkylating agent used for breast cancer therapy (Casanova et al., 2012). It was found that RES could be able to enhance the antitumor effects of MEL in MCF-7 and MDA-MB-231 cells. Interestingly, sequential treatment with RES followed by MEL yielded more cytotoxic effects than that treatment with MEL followed by RES. This effect may be related to the ability of RES to sensitize MCF-7 cells and MDA-MB-231 cells to MEL induced apoptosis. Similarly, Jazirehi and Bonavida (2004) reported that RES could sensitize NHL and MM cell lines to paclitaxel-mediated apoptosis, thus enhancing the antitumor efficacy of paclitaxel.

Rapamycin and its derivatives are potent inhibitors of dysregulated mTORC1 which can inhibit the proliferation of numerous tumor cell lines and tumor models. It was observed by He et al. that under combination with RES, the rapamycin-induced AKT phosphorylation was significantly decreased, and the antitumor activity of rapamycin in breast cancer cell lines was enhanced, suggesting that RES could assist rapamycin to increase the therapeutic efficacy (He et al., 2011).

Additionally, in another example, Lee et al. (2013) investigated the efficacy of the combined action of RES and clofarabine in malignant mesothelioma MSTO-211H cells. By calculating the CI, it was found that this combined treatment could produce a strong synergistic antiproliferative effect in MSTO-211H cells. RES was speculated to participate in the PI3K/AKT signaling pathway through reducing the content of Sp1, and the synergistic antitumor effect might be partly through the inhibition of AKT phosphorylation and the suppression of Sp1 activation.

### Mechanisms for RES-Medicated Cancer Therapy

**Figure 3:**
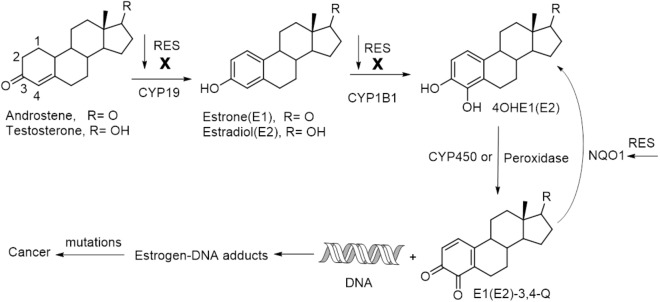
A simplified pathway for estrogens-induced cancer formation and RES-interfered cancer initiation.

As a naturally occurring small molecule, RES has been shown to be capable of mediating cancer therapy via targeting multiple pathways involving cancer initiation, promotion and progression (Elshaer et al., 2018). Cancer initiation is the first phase in cancer development, and a critical biomarker for this event is the elevated level of estrogen-DNA adducts in tissue, which indicates a high risk of cancer, such as in the etiology of breast cancer and prostate cancer (Cavalieri and Rogan, 2010; Zahid et al., 2011; Pruthi et al., 2012). RES can inhibit activating enzymes such as CYP19 (aromatase) and CYP1B1 (a kind of cytochrome P450 enzyme), and induce the expression of detoxification enzyme of NQO1 (NAD(P)H:quinone oxidoreductase 1), thus blocking the formation of estrogen-DNA adducts to protect against estrogen-initiated cancer (Figure 3). The synergistic effect of RES-mediated chemotherapy is also partly attributed to the interfering action to cancer initiation. In addition, RES-induced cell sensitization and the participation of RES in the modulation of cell cycle, especially in S –phase, also play significant roles for its synergistic effects (Jazirehi and Bonavida, 2004; He et al., 2011; Casanova et al., 2012; Lee et al., 2013).

**Figure 4:**
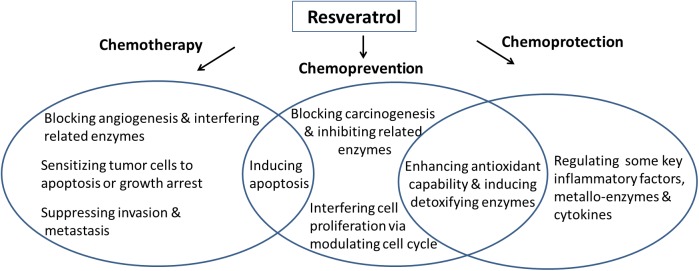
Potential mechanisms for RES-medicated chemotherapy, chemoprevention and chemoprotection.

RES is capable of inhibiting angiogenesis and metastasis through its interaction with multiple molecular targets. Kundu and Surh (2008) summarized the potential molecular targets of RES, and the mechanisms for RES-mediated cancer therapy modified from Kundu and Surh are shown in Figure 4 (Kundu and Surh, 2008). Blockade of angiogenic and metastatic process of cancer progression and reduction of the resistance of chemotherapy suggest the chemotherapeutic potential of RES. The chemopreventive potential of RES is reflected by its capability of blocking the activation of various carcinogens and/or to stimulate their detoxification, to avert oxidative damage to the target cells, to decrease inflammatory responses, and to reduce proliferation of cancer cells. The chemoprotective effects of RES are indicated by its anti-oxidizing property for scavenging ROS and improving the activities of some anti-oxidizing enzymes. RES is capable of activating NF-κB (nuclear factor κB) and SIRTI that are the major regulators for inflammatory response, and inhibiting some critical cytokines such as pro-inflammatory molecules of IL-1β, TNFα and IL-6, and oxidoreductase such as CBR, AKR and COXs. Besides, RES-mediated chemoprotection can also be achieved by reducing the expression of EMMPRIN-an important regulator for the synthesis of matrix metalloproteinase (MMP) to transport metal ions (Liu et al., 2016; Xiao et al., 2016). Overall, numerous mechanisms are involved for contributing to RES’s activity against cancerous and precancerous cells, and more studies and clinical trials are needed to examine RES’s efficacy in cancer therapy.

## Discussion and Future Perspective

In spite of tremendous efforts having been spent in searching efficient cures for cancer, cancer is still one of the greatest killers to human life. The incidence of cancer is rapidly increasing, and the number of cancer-related deaths is predicted to be double in the next 50 years. Although cancer chemotherapy is still currently one of the most frequently used treatment to combat cancer, it can only improve the overall survival of patients, while the quality of life is decreased. Moreover, chemotherapy often brings risks of chemo-resistance and easy recurrence as well as a variety of side effects including cardiotoxicity, nephrotoxicity and gastrointestinal toxicity. Due to the heterogeneity of cancer, it is also difficult to find a specific target for cancer therapy. As a consequence, the combinational treatment with multiple targets is regarded as one of the most promising approach to defeat cancer.

Natural products as one of the important resources in drug research and development, demonstrate a broad range of biological activities for the prevention, protection and treatment of different diseases. RES as a natural polyphenol is widely distributed in many types of foods. Using RES for the treatment and prevention of various diseases, especially for cancers, are extensively investigated. It has been proved that RES is able to interact with multiple molecular and cellular targets in cancer. The current associated data, both *in vitro* and *in vivo*, showed positive results in chemoprotective as well as synergistic aspects, suggesting that RES has great potential in the management of cancer chemotherapy. The lack of side effects is probably attributed to the low or moderate doses of RES that would be rapidly metabolized to the safe glucoronate and other analogs. In addition to investigations with high doses of RES in similar experiments, it is also imperative to perform some associated clinical trials needed for verifying these promising results. However, as searched from the database of human clinical studies on website of Clinical Trials.gov, there are a total of 151 records. Most of these trials are related to evaluate the safety, bioavailability, pharmacokinetics and tolerability of RES. Only a small amount of studies are focused on investigating the efficacy of RES in certain types of cancers. Up to now, there are no completed or ongoing clinical trials of RES in combination with other anticancer drugs for reducing the associated-side effects and enhancing the therapeutic outcomes in cancer chemotherapy. Numerous studies are primarily focused on the *in vitro* and *in vivo* animal experiments. It is encouraging that researchers can get some useful information and learn lessons from previous clinical trials while designing new trials, which will hopefully lead to better results.

Additionally, due to the low bioavailability of RES, further modification and optimization of RES for finding improved analogs with high bioavailability and good specificity are extremely important. For example, HTS has been used to screen and identify the specific and targeting peptide ligands against different cancer cells or disease biomarkers. If targeting peptides could be conjugated to RES against the expected cancer cells or the important proteins of interest, it is expected that it could greatly improve the efficacy of RES but lower down the side effects (Gray and Brown, 2013; Gao et al., 2016; Jee et al., 2016; Ong et al., 2017).

## Conclusion

To sum up, RES has been found to be capable of modulating cancer therapy through targeting multiple molecular and signal pathways. It can reduce the associated-side effects in cancer chemotherapy, such as cardiotoxicity, hepatotoxicity, renal toxicity, gastrointestinal toxicity, UVR-induced skin cancer, and carcinogens-induced injuries. RES is meanwhile capable of enhancing the outcomes by in combination with other chemotherapeutic drugs. These promising results will motivate researchers to perform more studies on RES, including further clinical trials, detailed mechanism investigations, and developing new derivatives with improved properties on enhancing the chemoprotective and therapeutic effects.

## Author Contributions

QX searched relative articles, created the outline and drafted the manuscript. WZ and WF assisted in searching relative articles and drafting the manuscript. SL and AL re-checked the relative articles and revised the manuscript. JS, LG and CX supervised the manuscript writing and revised the manuscript.

## Conflict of Interest Statement

The authors declare that the research was conducted in the absence of any commercial or financial relationships that could be construed as a potential conflict of interest.

## Abbreviations

5-FU: 5-fluorouracil
AKR: aldo-keto reductases
AKT: protein kinase B
ANT: anthracycline antibiotics
APL: promyelocytic leukemia
CBR: carbonyl reductases
CDK: cyclin-dependent kinases
CI: combination index
COX: cyclooxygenase
CYP19: a kind of aromatase
CYP1B1: a kind of cytochrome P450 enzyme
DMBA: 7,12-dimethylbenz[a]anthracene
Dox: doxorubicin
DOXol: doxorubicinol
EMMPRIN: extracellular matrix metalloproteinase inducer
eNOS: endothelial nitric oxide synthase
ERs: estrogen receptors
HTS: high through-put screening
MEL: melphalan
MM: multiple myeloma
MMP: matrix metalloproteinase
mTORC1: mammalian target of rapamycin complex-1
NAcCys: *N*-acetylcysteine
NASID: non-steroidal antiinflammatory drug
NHL: non-Hodgkin’s lymphoma
NQO1: (NAD(P)H:quinone oxidoreductase 1)
PGs: prostaglandins
PI3K: phosphatidylinositol-3-kinase
RAH: RES-aspirin hybrid
RES: resveratrol
ROS: reactive oxygen species
ROSC: roscovitine
SIRT1: sirtuin (silent mating type information regulation 2 homolog) 1
SOD: superoxide dismutase
Sp1: specificity protein 1
UVR: ultraviolet radiation
